# The Art of Cupping, &c. &c. &c.

**Published:** 1831

**Authors:** 


The Art of Cupping, &c. &c. &c. By George Frederick Knox, Cupper to the Westminster Hospital, &c. &c. Octavo, pp. 68. London, 1831. Price Three Shillings.

In this age of books we need not be surprised that cupping has its share. One would imagine that it would be difficult now-a-days to fill a goodly octavo with a description of the art of cupping and of the matters connected therewith; yet so it is, that Mr. Knox has contrived to execute the task in a very respectable manner. The following quotation will convey an idea of the author's style, and of the description of information to be met with in the volume; this is all that we can afford to do.

"I am in the habit of holding in my hand as many glasses as are necessary for the operation, but three may be held conveniently: the one to be first applied between the index finger and thumb of the left hand, another in the palm of the hand confined by the little-finger, the third on the three middle fingers; the torch must of course be held between the fingers and thumb of the right hand. In applying the glasses they should each in turn be held in a slanting direction, very nearly approaching to the perpendicular; the torch should be introduced about two-thirds of its depth, and then quickly, but with an easy, not a jerking motion, withdrawn, and the glass applied. It should be applied, not with a sudden, violent impulse upon the skin, nor with too slow a motion, but suffered to drop, as it were, simultaneously with the withdrawal of the torch; and there is no necessity whatever to press the glass upon the skin; it but adds to the pain, and the mere application of the edges to the surface is sufficient. Perhaps the best plan for a beginner, or a person having little practice, is to let one edge of the glass, always on the upper side, rest upon the skin, whilst the opposite edge is a little elevated to allow of the introduction of the torch, and suffered to fall again as it is withdrawn. It is a common error to allow the torch to continue too long within the glass; its stay should be momentary, as the exhaustion is instantaneous, and if the torch be suffered to remain the light will be extinguished. The glasses, when first applied, should remain on about half a minute, or until the skin assume a reddish purple colour, indicative of the blood being collected on the surface: and here comes the part of the operation requiring most dexterity. The torch should now be held in the palm of the right hand, and to avoid the liability of its being knocked away by the springing back of the trigger, with the upper portion of the tube pressing against the concavity formed between the fore-finger and thumb, and confined by the little and fourth fingers pressing upon the ring or plate—the scarificator should then be held between the thumb and middle-finger, care being taken not to press too soon upon the button—the fore-finger being at liberty, the nail should be gently introduced between the edge of the glass and the skin, pressing slightly inwards, and the glass gently elevated and taken off in the left hand; the scarificator should then be applied immediately before the subsidence of the tumour, flatly upon its surface; and upon the rapidity with which this is done depends, in great measure, the suffering of the patient; the instrument should then be placed in the palm of the left hand, confined by the little-finger, and the glass re-applied; and thus with each in succession, the glasses being repeated twice or thrice, according to the quantity of blood required, *over the same scarifications*. And here I cannot too strongly condemn the practice, as unnecessary as it is painful, and ghastly in appearance, of making two scarifications intersect each other; indeed, patients once treated in this manner will rarely give an opportunity of being so served a second time."

We recommend the work to those country surgeons who have had few opportunities of seeing the operation performed, and yet wish to become adepts in its arcana.

---

